# Community Opioid Dispensing After Injury (CODI): Protocol for a Population-Based Data Linkage Study

**DOI:** 10.2196/36357

**Published:** 2022-04-12

**Authors:** Cate M Cameron, Victoria McCreanor, Rania Shibl, Tanya Smyth, Melanie Proper, Jacelle Warren, Kirsten Vallmuur, Natalie Bradford, Hannah Carter, Nicholas Graves, Bill Loveday

**Affiliations:** 1 Jamieson Trauma Institute Royal Brisbane and Women's Hospital Metro North Health Brisbane Australia; 2 Australian Centre for Health Services Innovation and Centre for Healthcare Transformation Queensland University of Technology Kelvin Grove Australia; 3 School of Science Technology and Engineering University of the Sunshine Coast Petrie Australia; 4 Royal Brisbane and Women's Hospital Metro North Health Herston Australia; 5 Centre for Healthcare Transformation Queensland University of Technology Kelvin Grove Australia; 6 Health Services & Systems Research Duke-NUS Medical School Singapore Singapore; 7 QScript Management Unit Queensland Health Brisbane Australia

**Keywords:** opioids, injury, data linkage, cohort study, outcomes, epidemiology, population-based study, health records

## Abstract

**Background:**

There is an urgent need to reduce preventable deaths and hospitalizations from prescription opioid harms and minimize the negative effect opioid misuse can have on injured individuals, families, and the wider community. Data linkage between administrative hospitalization records for injured patients and community opioid dispensing can improve our understanding of the health and surgical trajectories of injured persons and generate insights into corresponding opioid dispensing patterns.

**Objective:**

The Community Opioid Dispensing after Injury (CODI) study aims to link inpatient hospitalization data with opioid dispensing data to examine the distribution and predictive factors associated with high or prolonged community opioid dispensing among adults, for 2 years following an injury-related hospital admission.

**Methods:**

This is a retrospective population-based cohort study of adults aged 18 years or older hospitalized with an injury in Queensland, Australia. The study involves the linkage of statewide hospital admissions, opioid prescription dispensing, and mortality data collections. All adults hospitalized for an injury between January 1, 2014, and December 31, 2015, will be included in the cohort. Demographics and injury factors are recorded at the time of the injury admission. Opioid dispensing data will be linked and extracted for 3 months prior to the injury admission date to 2 years after the injury separation date (last date December 31, 2017). Deaths data will be extracted for the 2-year follow-up period. The primary outcome measure will be opioid dispensing (frequency and quantity) in the 2 years following the injury admission. Patterns and factors associated with community opioid dispensing will be examined for different injury types, mechanisms, and population subgroups. Appropriate descriptive statistics will be used to describe the cohort. Regression models will be used to examine factors predictive of levels and duration of opioid use. Nonparametric methods will be applied when the data are not normally distributed.

**Results:**

The project is funded by the Royal Brisbane and Women’s Hospital Foundation. As of November 2021, all ethics and data custodian approvals have been granted. Data extraction and linkage has been completed. Data management and analysis is underway with results relating to an analysis for blunt chest trauma patients expected to be published in 2022.

**Conclusions:**

Little is currently known of the true prevalence or patterns of opioid dispensing following injury across Queensland. This study will provide new insights about factors associated with high and long-term opioid dispensing at a population level. This information is essential to inform targeted public policy and interventions to reduce the risk of prolonged opioid use and dependence for those injured. The novel work undertaken for this project will be vital to planning, delivering, monitoring, and evaluating health care services for those injured. The findings of this study will be used to inform key stakeholders as well as clinicians and pain management services.

**International Registered Report Identifier (IRRID):**

RR1-10.2196/36357

## Introduction

Prescription opioids are responsible for a large proportion of the preventable mortality, morbidity, and health care burden in many countries. This is a global and escalating issue. Canada, the United States, and Australia have some of the highest per capita opioid consumption in the world [1,2]. In the United States, overdose deaths involving prescription opioids have significantly increased yearly from 1999 to 2017 and again in 2020 [3]. In 2020, at least 72% of all overdose deaths involved opioids [4].

The Australian Institute of Health and Welfare reports that, each day in Australia, there are almost 150 hospitalizations, 14 emergency department presentations, and 3 deaths involving harm from pharmaceutical opioids [2,5]. In Australia, there has been a 3-fold rise in deaths with opioids present from 2010 to 2019 [6]. In 2018, opioid deaths accounted for 64.5% of all drug-induced deaths in Australia [7]. While opioids are commonly prescribed to injured patients to treat pain, their continued use can be controversial as taking opioids for longer periods of time or in higher doses can increase the risk of addiction, overdose, and death [8]. Characteristics of initial opioid prescription including higher dosage, long-acting opioids, and duration of prescription [9,10], as well as individual patient characteristics such as anxiety, prior opioid exposure [11], and higher self-reported levels of pain [12], are associated with long-term opioid use. There is an urgent need to reduce these preventable hospitalizations and deaths and the negative effect they have on injured individuals, families, health care providers, and the wider community.

Data linkage between administrative hospitalization records for injured patients and community opioid dispensing provides an important opportunity to improve our understanding of opioid use and inform opioid use reduction and harm minimization measures. Through data linkage, we can map patterns and identify demographic and health predictors of high or long-term opioid dispensing and evaluate the feasibility of routine linking of hospital data into existing regulatory prescription medicine monitoring processes. In a new and unique contribution to this field of research, our study will link inpatient hospitalization data with community opioid dispensing data to examine community opioid dispensing among adults for 2 years following an injury-related hospital admission in Queensland, Australia. These data are vital to planning, delivering, monitoring, and evaluating health care services and are essential to inform targeted public interventions to reduce the risk of prolonged opioid use and dependence for those injured.

The overall study objective is to examine the distribution and predictive factors associated with high or prolonged community opioid dispensing among adults for 2 years following an injury-related hospital admission in Queensland. The specific aims are as follows: (1) determine the prevalence of community opioid dispensing following injury-related hospitalizations in Queensland; (2) examine the patterns of community opioid dispensing during the 2 years following an injury-related hospital admission among adults, in terms of frequency, quantity, time post injury, and geographic distribution, as well as for different injury types, injury mechanisms, and population subgroups; and (3) identify predictors of high-level and long-term community opioid dispensing in adults following an injury-related hospital admission, for different injury types, mechanisms, and population subgroups.

## Methods

### Study Design

This is a retrospective population-based cohort study of adults aged 18 years or older hospitalized with an injury in Queensland, Australia, using linkage of statewide hospital admissions, community opioid dispensing, and mortality data collections. We anticipate there will be approximately 150,000 patients included in the analyses.

### Study Population

Queensland is a large state in Northeastern Australia covering an area of 1,852,642 km^2^, with a total population of 5.2 million people in 2021 [13]. Injured patients often must travel large distances to specialist trauma care, and for rehabilitation and follow-up with pain management services.

### Data Sources and Linkage

This study will link and analyze data extracted from three Queensland data collections, which are as follows: (1) admitted patient data from the Queensland Hospital Admitted Patients Data Collection (QHAPDC), (2) community opioid prescription and dispensing data from the Queensland Monitoring of Drugs of Dependence System (MODDS), and (3) Queensland Deaths data, from the Registry of Births, Deaths, and Marriages (BDM). At the date of the data request, the most recent data available for linkage were up to and including December 31, 2017.

Linkage will be undertaken by the Statistical Services Branch within Queensland Health, which enables linkage services to be conducted in a secure environment, ensuring compliance with strict security, privacy, and confidentiality requirements [14]. Using their Master Linkage File, individuals are matched across multiple health-related data collections and registries in Queensland using probabilistic and deterministic techniques followed by clerical review to manually inspect uncertain matches in probabilistic linkage [14]. Each person is then assigned a unique patient ID, which is appended to each data set to aid in ongoing linkage.

The MODDS data collection system has never been used for any record linkage before. Statistical Services Branch will undertake the new linkage of MODDS using the abovementioned principles. Once the data linkage has been completed, the Master Linkage File will be able to use the appended unique patient IDs in the MODDS database to enable future research to be streamlined. However, a new real time online prescription monitoring system (QScript) was introduced in Queensland in 2021 and will contain the prescription monitoring data moving forward [15].

**Figure 1:**
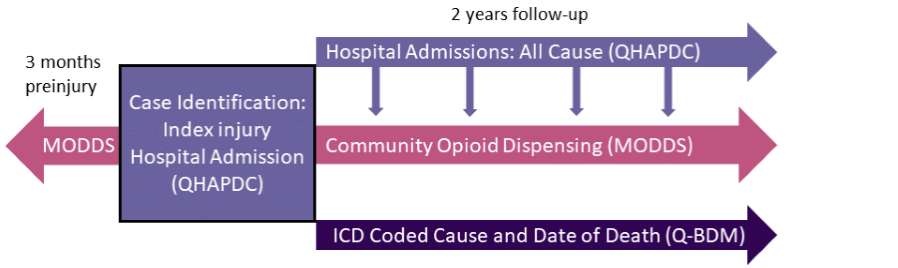
Community Opioid Dispensing After Injury (CODI) study design and data sources. ICD: International Classification of Diseases; MODDS: Monitoring of Drugs Dependence System; Q-BDM: Queensland Births, Deaths, and Marriages; QHAPDC: Queensland Hospital Admitted Patient Data Collection.

All adults hospitalized for an injury in Queensland, between January 1, 2014, and December 31, 2015, will be included in the cohort (Figure 1). The first injury-related hospital admission meeting the specified inclusion criteria described below will be identified as the “index injury admission.” Admission to hospital for any cause will be extracted for 2 years following the index injury separation (discharge) date. Opioid dispensing data for each injured person will be linked and extracted for the period 3 months prior to the index injury admission date through to 2 years following the index injury separation date. Deaths data will be linked and extracted for the 2-year follow-up period. The study covers a maximum period from October 1, 2013, to December 31, 2017.

### Hospital Admission Data: QHAPDC

Information collected on hospital admissions from the QHAPDC will include the hospital type (public or private) and health service area location; patient demographic details including age, sex, Aboriginal and/or Torres Strait Islander status, and area of residence. Patient admission details will include admission date, discharge date, care type, funding source, as well as International Classification of Diseases, 10^th^ Revision, Australian Modification (ICD-10-AM) diagnosis codes, Australian Classification of Health Interventions procedural codes, length of stay in an intensive care unit, and separation destination.

### Community Opioid Dispensing Data: MODDS

Community opioids dispensed for the cohort will be extracted from the MODDS data collection system. Medicines and poisons are classified into schedules according to the level of regulatory control over the availability of the medicine or poison required to protect public health and safety [16]. Opioids are included under Schedule 8 and are subject to monitoring in Queensland [16,17]. Schedule 8 are controlled drugs and are the highest level of control for prescription medicines.

Information extracted from the MODDS database will include the prescribed drug name, formulation, quantity prescribed, as well as prescribing and dispensing dates. Postcode of prescribing doctor and dispensing pharmacy will also be obtained for geographic analysis. The MODDS database also includes a classification code based on confirmed diagnosis of drug dependence or notifications of long-term treatment, which will also be extracted alongside prescription data.

### Mortality Data: Registry of Births, Deaths, and Marriages (BDM)

Mortality data for the injured cohort will be extracted from Queensland BDM including dates and cause of death. While the date of death is available in near real time, International Classification of Diseases (ICD)-coded cause of death is only available to Queensland Health when it has been processed by the Australian Bureau of Statistics. Coded cause of death data can take several years to become available, and until that time the cause of death is uncoded, but date of death is available.

### Cohort Inclusion and Exclusion Criteria

The cohort will be identified from QHAPDC. Subjects will be included if they meet all the following criteria: aged 18 years and over; admitted to any Queensland hospital for injury-related acute care between January 1, 2014, and December 31, 2015 (all public and private hospitals will be included); and had a principal diagnosis from ICD-10-AM codes, S00-T98.

After extraction, cohort subjects will be excluded from the analysis if their place of residence is outside Queensland, or they died during index injury admission. Non-Queensland residents would be lost to follow-up because our data are for Queensland only, and patients dying in hospital will not have subsequent prescriptions.

### Data Management and Classifications

#### Admitted Patient Data

It is possible for an individual to have multiple related or unrelated hospital admissions over the study period, so data sequencing logic is needed to distinguish records as (1) part of the same hospitalization (eg, a transfer); (2) a readmission or follow-up care related to the original index injury; (3) a new injury; or (4) a hospital admission for a reason other than injury. The study will apply data sequencing principles, logic, and definitions developed by Vallmuur et al [18], who use coded diagnoses, external causes, admission and discharge dates, transfer, and care type codes to distinguish between these records.

#### International Classification of Injury Severity Score (ICISS)

Injury severity for the index injury admission will be estimated using the International Classification of Injury Severity Score (ICISS), which is an ICD-based injury severity measure [19]. The ICISS is derived for each injured subject in the cohort by multiplying the probability of inpatient survival (ie, survival risk ratio) for each injury diagnosis (primary and all other diagnoses for the index injury admission). Using previously calculated survival risk ratios for Australia and New Zealand, ICISS can also be categorized into three severity categories: minor (≥0.99), moderate (>0.914 to <0.99), or serious (≤0.941) [20].

#### Accessibility/Remoteness Index of Australia

A measure of geographic remoteness is assigned to each subject in QHAPDC using their place of residence, mapped to the Accessibility/Remoteness Index of Australia [21]. Geographic areas are coded based on their road distance to service centers. Remoteness areas are classified as Major Cities, Inner Regional, Outer Regional, Remote, and Very Remote.

#### Socio-Economic Indexes for Areas (SEIFA)

A measure of socioeconomic status is assigned to each subject in QHAPDC using their place of residence and the Socio-Economic Indexes for Areas (SEIFA), developed by the Australian Bureau of Statistics [22]. SEIFA ranks areas in Australia according to relative socioeconomic advantage and disadvantage. The indices are based on information from the National five-yearly Census. In QHAPDC, SEIFA deciles are ranked from 1st decile for least advantaged through to the 10th decile for most advantaged.

### Opioid Data

To enable comparisons across opioid prescriptions of different types, formulations, and strengths, prescription data will be converted to oral morphine equivalents (OME). The OME of a drug is the dose of oral morphine that would produce the same level of pain relief. OME is the preferred method for analysis of opioid use for research purposes because it adjusts for the difference in potency across available types and formulations of opioids, via conversion factors [2]. The conversion factors available from the Australian and New Zealand College of Anaesthetists Faculty of Pain Medicine will be used for this study [23].

### Person-Years at Risk

A study end date will be created for all individuals in the cohort. The end date will be either the registered date of death, or if no death record exists, an individual will be deemed alive and therefore censored at the end of their 2-year follow-up period. Person-years at risk during the study period will be calculated individually as the time between the index injury separation date and the study end date.

### Ethics Approval

Ethics approval and a waiver of consent was obtained from the Royal Brisbane and Women’s Hospital Human Research Ethics Committee (HREC/2018/QRBW/48236). Additional approval was obtained for the release of confidential information for the purposes of research under the provision of Section 280 of Public Health Act 2005, Queensland (QCOS/033343/RD007954). As part of this Public Health Act application, the data custodians of each data source (QHAPDC, MODDS, and BDM) were required to provide authorization for use of the data source. No patients, prescribers, or dispensers of opioids will be identifiable in this study, and only nonidentifiable data will be obtained.

### Analysis Plan

Data analyses will be conducted using SAS 9.4 (SAS Institute). Appropriate descriptive statistics (frequencies, percentages, means, medians, standard deviations, and interquartile ranges) will be used to describe the injured cohort in terms of demographics, distribution of injury types, and mechanisms. Demographic and injury factors will be based on the characteristics recorded at the time of the index injury admission. Parametric and nonparametric tests will be used depending on the normality of the data.

While there are no agreed clinical guidelines for classifying opioid misuse, consideration will be given to classification of levels of opioid use based on total OMEs and duration or frequency of dispensing, informed by a review of the literature and applied consistently across the cohort. Regression models to examine demographic and abovementioned admitted patient factors predictive of levels and duration of opioid use will be applied. The opioid dispensing outcome data is not expected to be normally distributed. Logistic regression will be used for binary outcomes (eg, prolonged opioid use, yes/no), and either Poisson or Negative Binomial regression for counts of scripts and total OMEs, with an exposure time offset based on individual person-years at risk. Multinomial logistic regression will be used to examine opioid dispensing patterns, dependent on data and model parameters.

## Results

As of November 2021, all ethics and data custodian approvals for the study have been granted. This included approval under the Queensland Public Health Act 2005 for the release of patient information. Data extraction and linkage has been completed by the Statistical Services Branch. Deidentified data have been provided to the research team. Data management and analysis is underway. The first results relating to an analysis of opioid dispensing patterns following blunt chest trauma are expected to be published in 2022, with subsequent papers planned.

## Discussion

### Findings

Little is currently known of the true prevalence or patterns of opioid dispensing following injury in Queensland. While we know there is a significant escalation in opioid dispensing in Australia and worldwide [24,25], we do not know if there is an escalation for injured persons, or the demographic, injury, and health factors that may contribute to high or long-term use, or how frequently this occurs. Information about patterns of prescription opioid use beyond the acute in-hospital phase following injury is needed to inform policies and to enable targeted interventions to address pain management and reduce harmful opioid use.

The data in MODDS provide information on all public and private opioid dispensing in the community across the state. It will show frequency and quantity of dispensing and duration. However, there is no health information in MODDS around the circumstances for prescribing. Understanding the health circumstances for opioid prescribing is important for appropriate interpretation of individual opioid use. Many people who are hospitalized for an injury experience repeat admissions in the months or years following the injury, which may include multiple surgical procedures that can be planned or unplanned [26,27], all of which may necessitate intervals of opioid analgesia. Additionally, independent health conditions may develop (eg, cancer) that also warrant opioid analgesia and need to be identified to avoid attributing opioid patterns to the injury event, rather than other comorbidities. Thus, linking all-cause hospitalization data longitudinally can help to better map the health and surgical trajectories of injured persons and provide insights to the corresponding opioid dispensing patterns during the same period.

No previous research projects have linked to the MODDS data set. Without this data linkage, the new information generated in this project would require a lengthy, costly, and logistically challenging prospective cohort study, individually following up thousands of injured people in Queensland over several years. The most recent data available for linkage at the time of the current study data requests were December 31, 2017. Since that time, there have been more proactive strategies for opioid stewardship at varying stages of implementation in many countries, including Queensland, Australia [28]. Such strategies are aimed to reduce prescribing and provide alternative pain management and monitoring for patients. While this study will provide important information for a particular point in time, it will be important to revisit similar data systems beyond the time period of this study for surveillance purposes to identify any changes in long-term trajectories of opioid dispensing for injured populations. The Opioid Advisory Committee of Queensland will provide expert guidance on the data interpretation, which will be an invaluable additional external review process.

### Limitations

This is a retrospective observational study and will be limited to interpretations of association rather than causation. The data will be drawn from a defined period, and it will not be known if other societal factors or changes in the broader context of opioid prescribing in the same period may have influenced the results. The study sample is limited to persons hospitalized in Queensland; it will capture public and private hospitals but will not include injury-related presentations to emergency departments if patients are not admitted to the hospital, or injuries seen by general practitioners or community medical clinics.

There is no measure of pain severity in administrative patient data collections, and it will not be known if the opioid medications dispensed were administered in the prescribed manner by the person prescribed to. However, the data collection is statewide, and small sample deviations are unlikely to substantially affect population outcomes. Our data will not include some codeine-containing products that were available over the counter (Schedule 3) prior to 2018, or those in Schedule 4. All codeine preparations are now only either Schedule 4 or 8 medicines, and under new regulatory arrangements, are now included in prescription monitoring systems [25,29].

The linkage will not include hospitalization data prior to the study period, and therefore some people may have experienced prior injuries or previous hospital admissions that may have influenced opioid dispensing and will not be captured in the study. However, ongoing health issues are likely to be identified in the hospitalization database across the study period via primary and other diagnostic codes.

Although administrative data lack complex details on individual risk factors, they enable measurement of health service use longitudinally and comprehensively for the whole cohort. It will not be possible to identify persons who leave the state of Queensland; however, small sample deviations are unlikely to substantially affect population outcomes. While the quality of the Queensland Health linkage and data is considered high and manual clerical reviews are conducted, probabilistic and deterministic record linkage will always result in some false matches and mismatches; thus, the potential for error must be recognized [14].

### Conclusion

This study will provide a comprehensive statewide profile of opioid dispensing for 2 years following an injury-related hospitalization. It will provide new insights about factors associated with high or long-term opioid dispensing, including patient demographics, injury types, injury causes and severity, as well as comorbidities and subsequent admissions and procedures. This information will aid planning and monitoring health care service provision and inform harm reduction interventions for injured Queenslanders. This study will also inform the potential for routinely incorporating additional health data into the newly implemented real time monitored drug reporting system in Queensland to identify opportunities to reduce preventable mortality and morbidity.

## Abbreviations

BDM: Births, Deaths, and Marriages
CODI: Community Opioid Dispensing after Injury
ICD-10-AM: International Classification of Diseases, 10th Revision, Australian Modification
ICD: International Classification of Diseases
ICISS: International Classification of Injury Severity Score
MODDS: Monitoring of Drugs Dependence System
OME: oral morphine equivalent
QHAPDC: Queensland Hospital Admitted Patient Data Collection
SEIFA: Socio-Economic Indexes for Areas
